# 6-Chloro-2,4-bis­(dimethyl­amino)-1,3,5-trimethyl­borazine

**DOI:** 10.1107/S1600536813002420

**Published:** 2013-01-31

**Authors:** Mark A. Rodriguez, Theodore T. Borek

**Affiliations:** a PO Box 5800, MS 1411, Sandia National Laboratories, Albuquerque, NM 87185-1411, USA; b PO Box 5800, MS 0892, Sandia National Laboratories, Albuquerque, NM 87185-0892, USA

## Abstract

The borazine ring of the title mol­ecule, C_7_H_21_B_3_ClN_5_, shows a mild distortion from a planar to a flattened boat conformation. Steric effects due to the methyl and dimethyl­amine substituents appear to be the cause of this distortion.

## Related literature

The borazine ring in 2,4,6-tris­(dimethyl­amino)-1,3,5-trimeth­yl­borazine (Rodriguez & Borek, 2006 ▶) shows a greater distortion from planarity towards a boat conformation compared to the title compound. For the synthesis, see: Beachley & Durkin (1974 ▶).
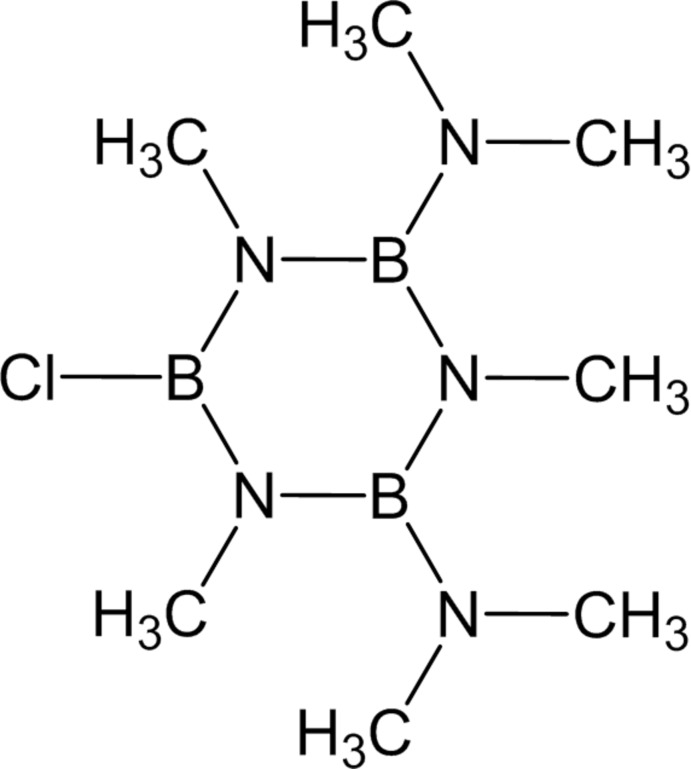


## Experimental

### 

#### Crystal data

C_7_H_21_B_3_ClN_5_
*M* *_r_* = 243.17
Monoclinic, 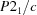

*a* = 8.493 (3) Å
*b* = 10.285 (3) Å
*c* = 15.247 (5) Å
β = 94.512 (4)°
*V* = 1327.8 (7) Å^3^
*Z* = 4
Mo *K*α radiation
μ = 0.27 mm^−1^
*T* = 193 K
0.25 × 0.20 × 0.15 mm

#### Data collection

Bruker APEXII CCD diffractometer
Absorption correction: multi-scan (*SADABS*; Bruker, 2005 ▶) *T*_min_ = 0.935, *T*_max_ = 0.962
9381 measured reflections
2397 independent reflections
1757 reflections with *I* > 2σ(*I*)
*R*_int_ = 0.037

#### Refinement

*R*[*F* ^2^ > 2σ(*F* ^2^)] = 0.040
*wR*(*F* ^2^) = 0.110
*S* = 1.03
2397 reflections
152 parameters
H-atom parameters constrained
Δρ_max_ = 0.18 e Å^−3^
Δρ_min_ = −0.23 e Å^−3^

### 

Data collection: *APEX2* (Bruker, 2005 ▶); cell refinement: *SAINT* (Bruker, 2005 ▶); data reduction: *SAINT*; program(s) used to solve structure: *SHELXTL* (Sheldrick, 2008 ▶); program(s) used to refine structure: *SHELXTL*; molecular graphics: *XSHELL* (Bruker, 2000 ▶); software used to prepare material for publication: *SHELXTL*.

## Supplementary Material

Crystal structure: contains datablock(s) global, I. DOI: 10.1107/S1600536813002420/lh5576sup1.cif

Structure factors: contains datablock(s) I. DOI: 10.1107/S1600536813002420/lh5576Isup2.hkl

Supplementary material file. DOI: 10.1107/S1600536813002420/lh5576Isup3.cml

Additional supplementary materials:  crystallographic information; 3D view; checkCIF report

## Figures and Tables

**Fig. 1.**
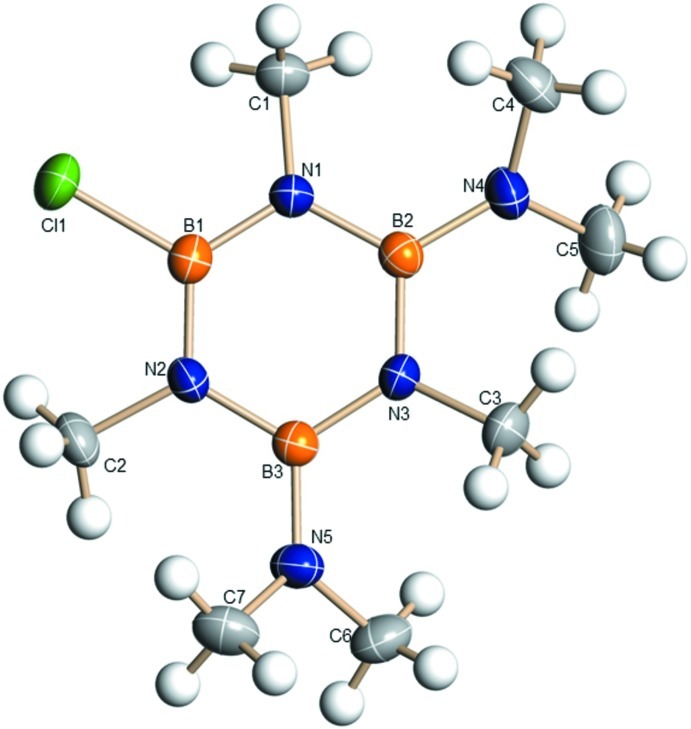
The molecular structure of (I), with 50% probability displacement ellipsoids for non-H atoms.

**Fig. 2.**
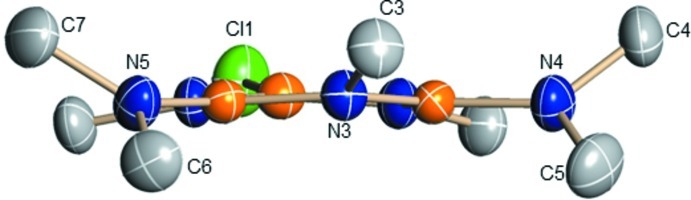
Side-view of molecule (I) to illustrate DMA rotations and boat-type borazine conform. H atoms have been removed for clarity. See text for details.

**Fig. 3.**
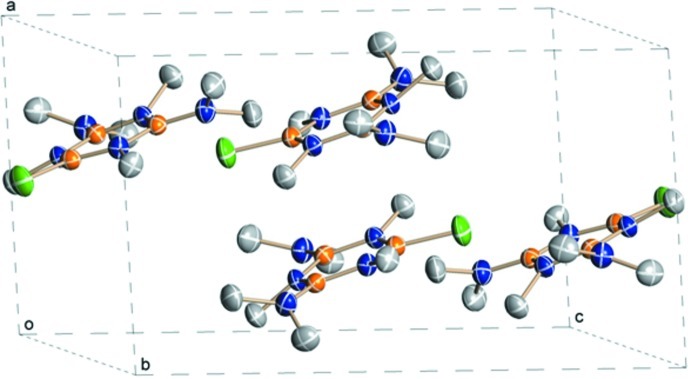
Packing diagram for (I) showing relative orientation of molecules in unit cell. H atoms have been removed for clarity.

**Fig. 4.**
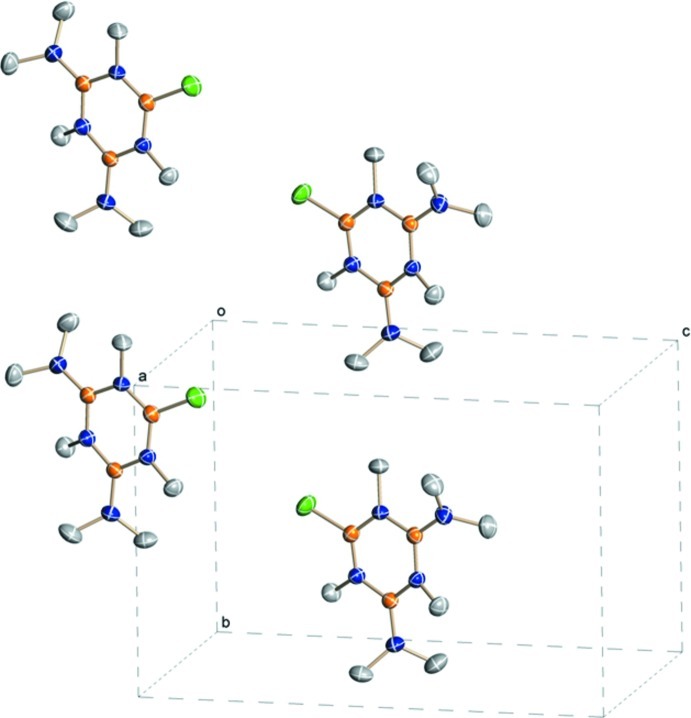
The effect of 21 screw-axis and c-glide plane symmetry elements which dictate molecule replication along the b axis. For clarity purposes H atoms have been removed as well as extra molecules.

## supplementary crystallographic information

### Comment

2,4-Bis(dimethylamino)-6-chloro-1,3,5-trimethylborazine (I) is a low melting point solid white material that has not been previously reported. Fig. 1 shows this molecule as an atomic displacement ellipsoid plot. All bond lengths for the dimethylamine (DMA) ligands, B—N bonds, and B—Cl bond are consistent with expected values. The heterogeneous nature of the ligands bound to the Boron atoms (one Cl and two DMA molecules) along with the steric nature of the DMA ligands with proximity to methyl groups bound to the nitrogen atoms of the borazine ring creates conditions in the molecule that drive the borazine ring away from a purely planar configuration. By defining the planar portion of the ring *via* the atoms N1/N2/B2/B3 and comparing the rotation of the DMA ligands away from the plane of the ring, dihedral angles may be obtained. For the case of the DMA ligand labeled with the N4 nitrogen (B2/N4/C4/C5 plane) the dihedral angle is 39.20 (7)° rotated out of borazine plane; the DMA ligand labeled with the N5 nitrogen (B3/N5/C6/C7 plane) has a dihedral angle of 37.25 (7)°, however it is rotated in the opposite direction. Fig. 2 shows an edge-on view of the molecule which better illustrates the dihedral rotation of the DMA molecules. Fig. 2 also serves to illustrate that the counter-rotations of the DMA molecules results in the C5 and C6 methyl groups being closer in proximity to the DMA-bracketed C3 methyl when compared to their respective DMA methyl counterparts residing above (in terms of the molecule orientation in the figure) the borazine plane (*i.e.* C4 and C7). The proximity of C5 and C6 methyl groups to C3 forces the C3 methyl to deviate, out of the plane of the borazine ring (N1/N2/B3/B2) by 16.5 (1)° (B2/B3/N3/C3). Figure 2 also illustrates that the Cl1 atom shifts slightly out of the borazine plane. This angular devation of the Cl1 atom is 6.38 (8)° (N2/B1/N1/Cl1) and the net result is a boat-type borazine configuration. The reduced severity of the Cl1 deviation from planar is likely due to the absence of bracketing DMA molecules. Instead, Cl1 is bracketed by methyl groups C1 and C2.

Figure 3 and 4 shows the packing arrangement in (I). Figure 3 illustrates the layering of the four molecules of (I) within the unit cell. Based on the long interaction distances between the terminal chlorine and the methyl hydrogen atoms of neighboring molecules, there does not appear to be significant hydrogren bonding interactions within this structure, and packing appears to be dictated by Van der Waals interactions. Figure 4 serves to illustrate the symmetry operators of the 2_1_ screw-axis and c-glide plane to replicate the molecule spatially along the *b* axis direction of the unit cell. In this figure, several molecules were removed for clarity.

### Experimental

Compound (1) was obtained using a modification of the published procedure of Beachley and Durkin (1974). One equivalent of 2,4,6-trichloro-1,3,5-trimethylborazine was reacted with 4 equivalents of anhydrous dimethylamine in anhydrous diethyl ether at room temperature. After stirring the reaction mixture overnight, the solution was filtered to remove precipitated dimethylammonium hydrochloride, and the solvent was removed using vacuum techniques. This product was then recrystallized from anhydrous hexanes, and then vacuum distilled (bp 355K at 270 mTorr). The liquid distillate slowly crystallized upon standing at room temperature resulting in a low melting point white solid with individual crystals displaying sufficient quality and size for single crystal structure analysis. The product purity was determined by nuclear magnetic resonance (1*H*, 11B, 13 C) and by gas chromatography/mass spectrometry.

### Refinement

H atoms were placed in calculated positions with C—H = 0.98Å and included in the refinement in a riding-motion approximation with U_iso_(H) = 1.5U_eq_(C).

### Crystal data

**Table:**

C_7_H_21_B_3_ClN_5_	*F*(000) = 520
*M**_r_* = 243.17	*D*_x_ = 1.216 Mg m^−^^3^
Monoclinic, *P*2_1_/*c*	Mo *K*α radiation, λ = 0.71073 Å
Hall symbol: -P 2ybc	Cell parameters from 200 reflections
*a* = 8.493 (3) Å	θ = 2.4–28.0°
*b* = 10.285 (3) Å	µ = 0.27 mm^−^^1^
*c* = 15.247 (5) Å	*T* = 193 K
β = 94.512 (4)°	Irregular, colorless
*V* = 1327.8 (7) Å^3^	0.25 × 0.20 × 0.15 mm
*Z* = 4	

### Data collection

**Table:**

Bruker APEXII CCD diffractometer	2397 independent reflections
Radiation source: fine-focus sealed tube	1757 reflections with *I* > 2σ(*I*)
Graphite monochromator	*R*_int_ = 0.037
ω and φ scans	θ_max_ = 25.3°, θ_min_ = 2.4°
Absorption correction: multi-scan (*SADABS*; Bruker, 2005)	*h* = −10→10
*T*_min_ = 0.935, *T*_max_ = 0.962	*k* = −12→12
9381 measured reflections	*l* = −17→18

### Refinement

**Table:**

Refinement on *F*^2^	Primary atom site location: structure-invariant direct methods
Least-squares matrix: full	Secondary atom site location: difference Fourier map
*R*[*F*^2^ > 2σ(*F*^2^)] = 0.040	Hydrogen site location: inferred from neighbouring sites
*wR*(*F*^2^) = 0.110	H-atom parameters constrained
*S* = 1.03	*w* = 1/[σ^2^(*F*_o_^2^) + (0.0507*P*)^2^ + 0.4074*P*] where *P* = (*F*_o_^2^ + 2*F*_c_^2^)/3
2397 reflections	(Δ/σ)_max_ = 0.001
152 parameters	Δρ_max_ = 0.18 e Å^−^^3^
0 restraints	Δρ_min_ = −0.23 e Å^−^^3^

### Special details

**Table:**

Geometry. All e.s.d.'s (except the e.s.d. in the dihedral angle between two l.s. planes) are estimated using the full covariance matrix. The cell e.s.d.'s are taken into account individually in the estimation of e.s.d.'s in distances, angles and torsion angles; correlations between e.s.d.'s in cell parameters are only used when they are defined by crystal symmetry. An approximate (isotropic) treatment of cell e.s.d.'s is used for estimating e.s.d.'s involving l.s. planes.
Refinement. Refinement of *F*^2^ against ALL reflections. The weighted *R*-factor *wR* and goodness of fit *S* are based on *F*^2^, conventional *R*-factors *R* are based on *F*, with *F* set to zero for negative *F*^2^. The threshold expression of *F*^2^ > σ(*F*^2^) is used only for calculating *R*-factors(gt) *etc*. and is not relevant to the choice of reflections for refinement. *R*-factors based on *F*^2^ are statistically about twice as large as those based on *F*, and *R*- factors based on ALL data will be even larger.

### Fractional atomic coordinates and isotropic or equivalent isotropic displacement parameters (Å^2^)

**Table:**

	*x*	*y*	*z*	*U*_iso_*/*U*_eq_	
B1	0.6753 (3)	0.9822 (2)	−0.09252 (15)	0.0322 (5)	
B2	0.7277 (3)	0.7848 (2)	−0.00232 (15)	0.0298 (5)	
B3	0.7837 (3)	1.0095 (2)	0.06221 (15)	0.0302 (5)	
Cl1	0.61969 (8)	1.05390 (6)	−0.19852 (4)	0.0508 (2)	
N1	0.66654 (19)	0.84528 (15)	−0.08386 (10)	0.0309 (4)	
N2	0.72169 (19)	1.06502 (15)	−0.02140 (10)	0.0308 (4)	
N3	0.79205 (19)	0.86955 (15)	0.06702 (10)	0.0311 (4)	
N4	0.7296 (2)	0.64530 (16)	0.00767 (12)	0.0386 (4)	
N5	0.8378 (2)	1.09064 (17)	0.13484 (11)	0.0380 (4)	
C1	0.5793 (3)	0.7691 (2)	−0.15374 (14)	0.0406 (5)	
H1A	0.4897	0.8202	−0.1793	0.061*	
H1B	0.5406	0.6885	−0.1288	0.061*	
H1C	0.6498	0.7481	−0.1997	0.061*	
C2	0.6864 (3)	1.20525 (19)	−0.02956 (15)	0.0412 (5)	
H2A	0.7721	1.2491	−0.0573	0.062*	
H2B	0.6767	1.2422	0.0290	0.062*	
H2C	0.5871	1.2175	−0.0658	0.062*	
C3	0.9084 (3)	0.8124 (2)	0.13352 (14)	0.0427 (6)	
H3A	0.9930	0.8753	0.1483	0.064*	
H3B	0.9532	0.7333	0.1097	0.064*	
H3C	0.8559	0.7907	0.1866	0.064*	
C4	0.7744 (3)	0.5554 (2)	−0.05884 (17)	0.0481 (6)	
H4A	0.8084	0.6041	−0.1093	0.072*	
H4B	0.6837	0.5005	−0.0780	0.072*	
H4C	0.8614	0.5005	−0.0344	0.072*	
C5	0.6986 (3)	0.5823 (2)	0.08946 (17)	0.0541 (7)	
H5A	0.7963	0.5433	0.1160	0.081*	
H5B	0.6189	0.5143	0.0778	0.081*	
H5C	0.6596	0.6466	0.1299	0.081*	
C6	0.8211 (3)	1.0560 (2)	0.22612 (14)	0.0490 (6)	
H6A	0.7483	0.9824	0.2285	0.074*	
H6B	0.7791	1.1305	0.2569	0.074*	
H6C	0.9244	1.0319	0.2545	0.074*	
C7	0.9317 (3)	1.2072 (2)	0.12748 (16)	0.0509 (6)	
H7A	0.9510	1.2215	0.0657	0.076*	
H7B	1.0328	1.1970	0.1624	0.076*	
H7C	0.8748	1.2819	0.1493	0.076*	

### Atomic displacement parameters (Å^2^)

**Table:**

	*U*^11^	*U*^22^	*U*^33^	*U*^12^	*U*^13^	*U*^23^
B1	0.0316 (13)	0.0337 (13)	0.0314 (12)	0.0012 (10)	0.0023 (10)	0.0042 (10)
B2	0.0303 (12)	0.0281 (12)	0.0314 (12)	−0.0014 (10)	0.0052 (9)	0.0012 (10)
B3	0.0298 (13)	0.0308 (12)	0.0304 (12)	−0.0014 (10)	0.0044 (10)	−0.0002 (10)
Cl1	0.0710 (4)	0.0453 (3)	0.0342 (3)	0.0001 (3)	−0.0081 (3)	0.0116 (3)
N1	0.0351 (10)	0.0279 (9)	0.0291 (9)	−0.0012 (7)	−0.0014 (7)	−0.0008 (7)
N2	0.0379 (10)	0.0227 (8)	0.0320 (9)	0.0005 (7)	0.0032 (7)	0.0012 (7)
N3	0.0348 (10)	0.0303 (9)	0.0273 (9)	0.0019 (7)	−0.0022 (7)	0.0037 (7)
N4	0.0491 (11)	0.0273 (9)	0.0396 (10)	−0.0002 (8)	0.0046 (8)	0.0046 (8)
N5	0.0463 (11)	0.0351 (10)	0.0328 (9)	−0.0062 (8)	0.0037 (8)	−0.0048 (8)
C1	0.0465 (13)	0.0369 (12)	0.0372 (12)	−0.0044 (10)	−0.0047 (10)	−0.0056 (10)
C2	0.0523 (14)	0.0250 (11)	0.0468 (13)	0.0051 (10)	0.0079 (11)	0.0027 (10)
C3	0.0453 (13)	0.0443 (13)	0.0370 (12)	0.0047 (11)	−0.0070 (10)	0.0059 (10)
C4	0.0518 (15)	0.0294 (12)	0.0631 (16)	0.0010 (11)	0.0034 (12)	−0.0060 (11)
C5	0.0604 (17)	0.0430 (14)	0.0589 (16)	−0.0044 (12)	0.0037 (13)	0.0200 (12)
C6	0.0556 (15)	0.0590 (15)	0.0318 (12)	−0.0033 (12)	−0.0009 (10)	−0.0053 (11)
C7	0.0521 (15)	0.0469 (14)	0.0536 (15)	−0.0107 (12)	0.0028 (12)	−0.0141 (12)

### Geometric parameters (Å, º)

**Table:**

B1—N2	1.410 (3)	C2—H2A	0.9800
B1—N1	1.417 (3)	C2—H2B	0.9800
B1—Cl1	1.805 (2)	C2—H2C	0.9800
B2—N4	1.443 (3)	C3—H3A	0.9800
B2—N3	1.444 (3)	C3—H3B	0.9800
B2—N1	1.450 (3)	C3—H3C	0.9800
B3—N5	1.433 (3)	C4—H4A	0.9800
B3—N3	1.443 (3)	C4—H4B	0.9800
B3—N2	1.457 (3)	C4—H4C	0.9800
N1—C1	1.474 (2)	C5—H5A	0.9800
N2—C2	1.476 (2)	C5—H5B	0.9800
N3—C3	1.481 (3)	C5—H5C	0.9800
N4—C4	1.446 (3)	C6—H6A	0.9800
N4—C5	1.448 (3)	C6—H6B	0.9800
N5—C7	1.449 (3)	C6—H6C	0.9800
N5—C6	1.454 (3)	C7—H7A	0.9800
C1—H1A	0.9800	C7—H7B	0.9800
C1—H1B	0.9800	C7—H7C	0.9800
C1—H1C	0.9800		
			
N2—B1—N1	122.81 (19)	N2—C2—H2C	109.5
N2—B1—Cl1	118.66 (16)	H2A—C2—H2C	109.5
N1—B1—Cl1	118.51 (16)	H2B—C2—H2C	109.5
N4—B2—N3	121.51 (19)	N3—C3—H3A	109.5
N4—B2—N1	121.18 (19)	N3—C3—H3B	109.5
N3—B2—N1	117.26 (18)	H3A—C3—H3B	109.5
N5—B3—N3	122.03 (19)	N3—C3—H3C	109.5
N5—B3—N2	121.31 (19)	H3A—C3—H3C	109.5
N3—B3—N2	116.64 (18)	H3B—C3—H3C	109.5
B1—N1—B2	119.26 (17)	N4—C4—H4A	109.5
B1—N1—C1	119.19 (17)	N4—C4—H4B	109.5
B2—N1—C1	121.17 (16)	H4A—C4—H4B	109.5
B1—N2—B3	119.74 (17)	N4—C4—H4C	109.5
B1—N2—C2	118.85 (17)	H4A—C4—H4C	109.5
B3—N2—C2	120.90 (17)	H4B—C4—H4C	109.5
B3—N3—B2	123.44 (17)	N4—C5—H5A	109.5
B3—N3—C3	117.28 (17)	N4—C5—H5B	109.5
B2—N3—C3	117.08 (17)	H5A—C5—H5B	109.5
B2—N4—C4	124.30 (18)	N4—C5—H5C	109.5
B2—N4—C5	122.33 (18)	H5A—C5—H5C	109.5
C4—N4—C5	113.18 (18)	H5B—C5—H5C	109.5
B3—N5—C7	124.53 (18)	N5—C6—H6A	109.5
B3—N5—C6	123.20 (18)	N5—C6—H6B	109.5
C7—N5—C6	111.87 (18)	H6A—C6—H6B	109.5
N1—C1—H1A	109.5	N5—C6—H6C	109.5
N1—C1—H1B	109.5	H6A—C6—H6C	109.5
H1A—C1—H1B	109.5	H6B—C6—H6C	109.5
N1—C1—H1C	109.5	N5—C7—H7A	109.5
H1A—C1—H1C	109.5	N5—C7—H7B	109.5
H1B—C1—H1C	109.5	H7A—C7—H7B	109.5
N2—C2—H2A	109.5	N5—C7—H7C	109.5
N2—C2—H2B	109.5	H7A—C7—H7C	109.5
H2A—C2—H2B	109.5	H7B—C7—H7C	109.5
